# Using electronic admission data to monitor temporal trends in local medication use: Experience from an Australian tertiary teaching hospital

**DOI:** 10.3389/fphar.2022.888677

**Published:** 2022-10-14

**Authors:** Richard J. Woodman, Chris Horwood, Aline Kunnel, Paul Hakendorf, Arduino A. Mangoni

**Affiliations:** ^1^ Centre of Epidemiology and Biostatistics, College of Medicine and Public Health, Flinders University, Adelaide, SA, Australia; ^2^ Department of Clinical Epidemiology, Flinders Medical Centre, Southern Adelaide Local Health Network, Adelaide, SA, Australia; ^3^ School of Mathematical Sciences, University of Adelaide, Adelaide, SA, Australia; ^4^ Discipline of Clinical Pharmacology, College of Medicine and Public Health, Flinders University, Adelaide, SA, Australia; ^5^ Department of Clinical Pharmacology, Flinders Medical Centre, Southern Adelaide Local Health Network, Adelaide, SA, Australia

**Keywords:** electronic hospital data, data management, temporal trends, medication usage, comorbidity, pharmacoepidemiology

## Abstract

**Background and aims:** Medication usage varies according to prescribing behavior, professional recommendations, and the introduction of new drugs. Local surveillance of medication usage may be useful for understanding and comparing prescribing practices by healthcare providers, particularly in countries such as Australia that are in the process of enhancing nationwide data linkage programs. We sought to investigate the utility of electronic hospital admission data to investigate local trends in medication use, to determine similarities and differences with other Australian studies, and to identify areas for targeted interventions.

**Methods:** We performed a retrospective longitudinal analysis using combined data from a hospital admissions administrative dataset from a large tertiary teaching hospital in Adelaide, South Australia and a hospital administrative database documenting medication usage matched for the same set of patients. All adult admissions over a 12-year period, between 1 January 2007 and 31st December 2018, were included in the study population. Medications were categorized into 21 pre-defined drug classes of interest according to the ATC code list 2021.

**Results:** Of the 692,522 total admissions, 300,498 (43.4%) had at least one recorded medication. The overall mean number of medications for patients that were medicated increased steadily from a mean (SD) of 5.93 (4.04) in 2007 to 7.21 (4.98) in 2018. Results varied considerably between age groups, with the older groups increasing more rapidly. Increased medication usage was partly due to increased case-complexity with the mean (SD) Charlson comorbidity index increasing from 0.97 (1.66) in 2007-to-2012 to 1.17 (1.72) in 2013-to-2018 for medicated patients. Of the 21 medication classes, 15 increased (*p* < 0.005), including antithrombotic agents; OR = 1.18 [1.16–1.21], proton pump inhibitors; OR = 1.14 [1.12–1.17], statins; OR = 1.12; [1.09–1.14], and renin-angiotensin system agents; OR = 1.06 [1.04–1.08], whilst 3 decreased (*p* < 0.005) including anti-inflammatory drugs (OR = 0.55; 99.5% CI = 0.53–0.58), cardiac glycosides (OR = 0.81; 99.5% CI = 0.78–0.86) and opioids (OR = 0.82; 99.5% CI = 0.79–0.83). The mean number of medications for all admissions increased between 2007 and 2011 and then declined until 2018 for each age group, except for the 18-to-35-year-olds.

**Conclusion:** Increased medication use occurred in most age groups between 2007 and 2011 before declining slightly even after accounting for increased comorbidity burden. The use of electronic hospital admission data can assist with monitoring local medication trends and the effects of initiatives to enhance the quality use of medicines in Australia.

## Introduction

The increasing availability of medicines represents a critical feature of modern patient care. Several studies have reported, particularly over the last 25 years, a temporal increase in the use of various drugs in different countries (Hollingworth et al., 2010; Sumukadas et al., 2014; Kantor et al., 2015; Gao et al., 2018). This phenomenon is largely attributed to the progressive ageing and the increasing medical complexity of the population as well as the practice of inappropriate prescribing and polypharmacy. Inappropriate prescribing and polypharmacy are significantly associated with the risk of drug-drug interactions and several adverse health outcomes, particularly falls, hospitalization, and death (Davies et al., 2020; Hoel et al., 2021; Mekonnen et al., 2021). More recently, associations have also been reported between polypharmacy and frailty and between the use of specific medications and the risk of cognitive and functional decline (Hilmer et al., 2009; Lowry et al., 2011; Bostock et al., 2013; Kouladjian et al., 2014; Gray et al., 2015; Ruxton et al., 2015; Gutierrez-Valencia et al., 2018). This has prompted several professional groups to develop recommendations to promote rational deprescribing interventions and ensure the quality use of medicines (Scott et al., 2015; Reeve et al., 2017; Farrell et al., 2019; Bloomfield et al., 2020).

Australia spends in excess of $20 billion on medicines annually. At the same time, studies have shown that medication misadventures account for 2–3% of hospital admissions. However, this figure increases to 20–30% in patients ≥65 years, with an associated cost in excess of $1.3 billion (Pearson et al., 2021). The capacity to monitor the use of medications at the population level, using ‘real-world’ data, represents one of the tenets of pharmacoepidemiology. Current Australian data sources available to investigate medication use primarily include dispensing records, electronically generated to record transactions and attract reimbursement for dispensing pharmacies (Pearson et al., 2015), self-reports as part of national surveys (Banks et al., 2008), specific disease registries (Lee et al., 2018), and surveillance systems for controlled drugs (Dobbin and Liew, 2020). However, available medication data from these sources remain largely unlinked from information regarding other patient characteristics, e.g., comorbidities, socioeconomic status and frailty, and clinical outcomes (Pearson et al., 2021). This curtails a comprehensive assessment of medication use across the population and the development of targeted educational interventions. Pending the implementation of robust state, territory, and federal data linkage strategies to capture medication use, the progressive roll-out of electronic systems that record hospital admission data over the last 15 years offers an alternative approach to investigate trends in medication use within local health networks. The overall aim of this study was to assess the utility of using electronic hospital admission data to investigate local trends in medication use, to determine similarities and differences with other Australian and international studies, and to identify areas for targeted interventions. More specifically, the primary aims were to 1) describe the changes in usage rates for 21 pre-specified drug classes over time, 2) describe the changes in total number of medications used per patient over time, 3) describe the changes in usage rates of the most listed medications amongst medicated patients over time, and 4) compare drug-class usage during the most recent 6-year time period with drug-class usage in the previous 6-year period. Secondary aims were to describe and compare changes in hospital admission rates and the Charlson comorbidity Index over time.

## Methods

### Study design

We performed a retrospective longitudinal analysis to describe trends in medication usage in patients admitted to a 593-bed metropolitan tertiary teaching hospital (Flinders Medical Center, Southern Adelaide Local Health Network, Adelaide, South Australia) with a catchment area of ∼350,000 people. Flinders Medical Centre serves as the trauma center for the Southern Adelaide region. More than 5,000 health care staff provide around-the-clock emergency care, intensive care, medical, surgical, obstetrics and gynecology, pediatric, and oncology services. The hospital is co-located with the Flinders University College of Medicine and Public Health. The calendar period of the study was from 1st January 2007 until 31st December 2018. The period of study was chosen to ensure complete availbility and consistency over time for both the hospital admissions and medications data at the time that the analysis was first commenced in mid-2019. The study population consisted of all adult admissions within this period and included all admissions and all medications at each admision for any individual patient. Admissions for patients less than 18 years of age were excluded from the analysis. We also performed a cross-sectional comparison study to assess the differences in medication usage, hospital admission rates and commorbidities between 2007–2012 and 2013–2018.

### Data sources

The data for hospital admissions were obtained from the Integrated South Australian Activity Collection (ISAAC) database. Variables extracted included patient identifier, admission date, age, gender, postcode, and primary and secondary diagnosis codes. We also constructed several additional variables including the Socio-Economic Index for Areas (SEIFA, using postcode), the Charlson comorbidity index (CCI, using secondary diagnosis codes), age categories (18–34 years, 35–49 years, 50–64 years, 65–79 years, and 80 + years of age), and study period (2007–2012 = Period 1 and 2013–2018 = Period 2). SEIFA is a product developed by the Australian Bureau of Statistics (ABS) ranks areas in Australia according to relative socio-economic advantage and disadvantage. It is generated using 5-year census data and based on an individual’s postcode of residence. The national overall mean value is 1,000 with higher values indicating relatively more advantage and lower numbers indicating relatively more disadvantage. The matching data for the medications recorded for the hospital admissions came from the Open Architecture Clinical Information System (OACIS) database. Matching was performed using the patient’s Unique Record Number (URN) and admission date. These datasets are available to researchers *via* the Clinical Epidemiology Unit of FMC upon obtainining the required permissions from the local Ethics review board. Variables extracted from OACIS included the patient identifier, admission date, and all medication names (as a typed string field). Upon admission to the hospital, and again at discharge from the hospital, a patient’s currently prescribed and newly prescribed medications respectively will be recorded by the treating clinician. Either the generic or tradename of the medication is typed into the appropriate recording field for each patient in the OACIS database. The medications included in this dataset included only those medications that the patient was taking upon admission to the hospital and did not include newly prescribed medications during the hospital stay. The medications data was matched with the hospital admissions data using the unique patient identifier in each database and the admission date. The resulting database had either 1 or multiple records for each admission date, depending on the number of medications recorded per patient (patients with no medications recorded had one record for each admission). Only medications that the patient was currently using at the time of admission were included for analysis.

### Categorization of the medications

Prior to analysis it was necessary to categorize the string typed medication names that included numerous different spellings of trade and generic medication names into a single common generic medication name. This was achieved with the aid of text mining software (WordStat version 9.0.5, Provalis Research, Montreal, Canada) which allows users to develop their own customised Categorisation Model to categorise text from documents or string-variable databases. We therefore developed a “Categorization Model” for the medications which enabled an automated categorization of the separte words contained in each of the 2,221,029 separate medication records in the OACIS medications database for the period of interest, into one of 1,095 different generic medication names. Initially, the WordStat software was used to scan the text within each of the separate medication records and provide a list of all the unique words. Any misspelt words are identified as being separate words. Words that are closely matched (i.e., with either only one or two misspellings) are automatically identified and can be merged together into the same folder (i.e., category) at the users discretion. We also identified and merged words which related to the same generic medication (but were a tradename of the medication) or were other misspellings, placing them into the appropriate folder. This categorisation process was initially performed using only data from 2012 to 2014. Once the initial categorisation dictionary had been created from these 3 years of data, the additional years of data were also processed using this previously defined categorisation dictionary. Any words not already classified by the initial dictionary were again, manually classified into the appropriate existing folder or into a new generic drug-name folder, thereby updating the categorisation model. After the final run of the data which included all 12 years of data, all words from the medication records were grouped into either one of the 1,095 different generic medication names of interest or placed into an exclusion list. This established the final categorisation model. We then used a Microsoft Excel spreadsheet to further categoise theis list of medications into 21 different drug classes of interest, according to the ATC code list 2021, covering a wide range of medical conditions. Any medications outside of these drug classes were included in an “Other” drug class category. The 21 different classes of drugs were proton pump inhibitors, diabetes, antithrombotic agents, cardiac glycosides, antiarrhythmics, vasodilators, diuretics, beta-blocking agents, calcium channel blockers, renin-angiotensin system agents, statins, corticosteroids, anti-inflammatory and antirheumatic agents, opioids, antiepileptics, anti-Parkinson drugs, psychotropics, antidepressants, anti-dementia drugs, obstructive airways diseases drugs, and antihypertensives (Supplementary Table S1).

### Categorisation of age and charlson comorbidity index

Age was categorised as (18–34 years, 35–49 years, 50–64 years, 65–79 years, and 80 + years of age). These age definitions have been used in pharmacoepidemiologic and health service research studies conducted in Australia and overseas (Hartmann et al., 2016; Black-Tiong et al., 2021). The continuous Charlson Comorbidity Index was categorised for frequency analysis according to the method suggested (Mild = 1 or 2, Moderate = 3 or 4, Severe = 5 or more) (Charlson et al., 1987).

### Data validation

As an indicator of the completeness of the captured hospital admission data, we confirmed that the total number of admissions recorded in the ISAAC database for each calendar year from 2011 to 2018 including patients <18 years old corresponded closely to that reported on the AIHW MyHospital website for Flinders Medical Centre for the Financial Years 2011–2018 (2011: 59,313 vs 58,424; 2012: 62,682 vs 60,393; 2013: 63,866 vs 63,441; 2014: 68,686 vs 65,176; 2015: 72,297 vs 71,665; 2016: 76,643 vs 73,273; 2017: 80,085 vs 82,509; 2018: 83,733 vs 82,263). For the medications data we used a random sample of 1,000 medication records and checked the accuracy of the original string medication variable with the newly created variable that contained only the generic medication name. All 1,000 records were correctly classified in accordance with the generic medications’ classification dictionary.

### Statistical power

A retrospective statistical power analysis was performed for the width of the 95% confidence interval for the prevalence of drug class use, and the odds ratios for drug class use between periods 1 and 2 of the study, the two primary outcomes of interest. The largest width for a 95% confidence interval for prevalence was obtained by calculating the width for the lowest estimated drug class usage for any 1 year. For odds ratios, the detectable effect size with 80% power was calculated for classes with a prevalence of either 0.5% or 30%, assuming a nominal 20,000 admissions per year and a 2-sided Type-1 error rate of alpha = 0.05. The lowest estimated prevalence of use for any of the drug classes in any given year was 0.33% (82/24,707) for anti-dementia drugs. This provided a 95% confidence interval with a width of 0.147% (95% CI = 0.262%–0.410%). The study had 80% power to detect an odds ratio of 1.17 or more for drug classes between periods, assuming a class prevalence of as small as 0.5% in one period and a minimum of 20,000 adult admissions with recorded medications each year (i.e., 120,000 per period). The study also had more than 80% power to detect an odds ratio of 1.03 or more for drug classes with a higher usage prevalence of 30%, and a minimum of 20,000 recorded adult medication admissions per year.

### Statistical analysis

The frequency of hospital admissions in each period was described using frequencies and percentages for each of the 5 age groups. To describe changes over time for drug-class use we used line plots with the calendar year as the unit of time. In addition, we also divided the study period into 2 equal lengths (2007–2012 and 2013–2018) to allow us to calculate odds ratios for drug class use for the second 6-year period compared to the first 6-year period. The line plots provide a description of the functional form for the association between usage and time whilst the odds ratios provide a more global summary of the overall change across time for each of the 21 drug classes. Given the large sample size for our study we set the nominal confidence type 1-error rate to alpha = 0.01 and used 99% confidence intervals for our overall comparisons. However, for the age-group comparisons in which we had 5 times as many comparisons and therefore a greater risk of a type 1 error rate we set the type 1 error rate to 0.01/5 = 0.002 and used 99.5% confidence intervals based on a Bonferroni method of correction for multiple comparisons. For the comparison of drug classes in which there were approximately 20 comparisons of interest we set the Type 1 error rate to 0.01/20 = 0.0005 and used 99.9% confidence intervals. Differences between periods in overall admission rates, medicated admission rates, and non-medicated admission rates were assessed using negative binomial regression with robust standard errors. Negative binomial regression models are appropriate for count data in the presence of overdispersion, where the variance is greater than the mean of the distribution and cannot therefore be considered a Poisson distribution (Harris et al., 2014). A likelihood ratio test was used to check for the model assumption of overdispersion of the mean. The study period (1 vs 2) was the independent variable, and results were described using incidence rate ratios (IRR) with 95% confidence intervals. The Stata command “nl” was used to fit an arbitrary nonlinear regression function by least squares to determine the month and year of changes in the linear trends of mean medication usage. Non-linear regression fits arbitrary separate regression lines to the data using least-squares in a piecewise fashion, with the number of lines fit being dependent on the data. Prior to analysis, the approximate coordinates at which there is a change in slope of the piecewise function are provided as starting points based on a visual inspection of the data. The exact points at which the regression line changes slope is then determined by the regression model. The process is very similar to join-point regression and has also been found to be superior to other non-linear estimation methods (Ratkowsky, 1983). For this analysis, admissions were aggregated by month and the analysis was performed with and without adjustment for age, sex, month, and the grouped CCI (0/1/2). Poisson regression with restricted cubic splines was used to describe the functional form of the relationship between the mean number of medications per admission and calendar month and year for each of the 5 age groups. Analysis was adjusted for sex, month, and the grouped CCI (0/1/2). Differences in gender between periods for the medicated and for non-medicated admission rates were assessed using logistic regression with robust standard errors. Gender was the dependent variable and period was the independent variable. The analysis was stratified by age group. Analysis was performed using Stata (StataCorp, version 17.0, College Station, TX, USA). Graphics were produced using Stata except for the plot of drug class prevalence which was produced using Python (version 3.9.7, Wilmington, DE, USA) with the matplotlib and dufte packages.

## Results

### Linkage of hospital and medication data

Supplementary Figure S1 describes the number of hospital admissions and the number of medications extracted within the study period from the ISAAC and OACIS databases respectively. The flow arrows outline the removal of admissions and medications due to either patient exclusion criteria (<18 years of age) or non-coded medications. The resulting number of admissions, medications, and their corresponding drug clases that were used in the analysis are also described.

### Patient characteristics

Supplementary Table S2 describes the study population within each study period in terms of age, gender, CCI, SEIFA and Primary diagnoses. There were no missing data for age, gender, the CCI or length-of-hospital stay (days) for either period. There were 673 missing values for SEIFA (obtained from postcode) for period 1 and 990 missing values for SEIFA in period 2. The median (IQR) for age in period 1 was 60 (40–76) and for period 2 was 62 (40–77) (*p* < 0.001). Of the 304,378 admissions in period 1, there were 144,466 (47.5%) males and 159,912 (52.5%) females. Of the 388,144 admissions in period 2, there were 182,044 (46.9%) males and 206,100 (53.1%) females (*p* < 0.001). The mean (SD) SEIFA score in period 1 was 981.9 (63.1) and in period 2 was 981.5 (62.1) (*p* < 0.001). In period 1 there were n = 3,781 (1.24%) deaths and in period 2 there were 4,037 deaths (1.04%). In period 1, 3,008 admissions (0.99%) resulted in a discharge to a residential aged care facility compared to 3,156 (0.81%) in period 2 (*p* < 0.001). According to the listed primary and secondary diagnoses for each admission, the prevalence of infectious disease, hepatitis B, endocrine disease, respiratory disease, digestive disturbances, rheumatic disease and chronic renal failure all increased between period 1 and period 2 (*p* < 0.001 for each), whereas the prevalence of malignancy, use of marijuana, dementia/alzheimers, cardiovascular disease all decreased (*p* < 0.001 for each).

### Hospital admission rates

Table 1 shows the total number of admissions and daily admission rates to Flinders Medical Centre for each of the 5 age groups and each of the 2 study periods. Across the 12 years of the study (2007–2018), there were a total of 692,522 adult admissions. A total of 304,378 occurred between 2007 and 2012 (Period 1) and 388,144 occurred between 2013 and 2018 (Period 2). The largest percentage of admissions came from the 65-to-79-year-old age group which contributed 25.15% of admissions with a mean (SD) admission rate of 39.2 (15.3) admissions per day. This was 50% higher than the admission rate for the 35–49-year-old age group that had a mean (SD) admission rate of 25.9 (9.5) admissions per day (IRR = 1.50; 95% CI = 1.40, 1.61; *p* < 0.001). The admission rates for 2013–2018 were higher for all age groups than the admission rates for 2007–2012, with the largest increase being for the 65–79-year-olds (IRR = 1.42; 95% CI = 1.39, 1.44; *p* < 0.001).

**TABLE 1:** Number of hospital admissions by age-group and study period.

	All years	Period 1	Period 2	
	2007–2018	2007–2012	2013–2018	
	N = 692,522	N = 304,378	N = 388,144	
Total admissions^a^	N (%)	N (%)	N (%)	Age-group vs 35–49 IRR (95% CI)^a^
18–35 years	129,687 (18.7)	57,405 (18.9)	72,282 (18.6)	1.14 (1.06, 1.21)<0.001
35–49 years	113,558 (16.4)	52,564 (17.3)	60,994 (15.7)	1.00 (Reference group)**-**
50–64 ears	143,416 (20.7)	64,731 (21.3)	78,685 (20.3)	1.26 (1.17, 1.36)*p* < 0.001
65–79 years	97,611 (25.15)	74,166 (24.4)	97,611 (25.15)	1.50 (1.40, 1.61)<0.001
80 + years	78,572 (20.2)	55,512 (18.2)	78,572 (20.2)	1.17 (1.05, 1.29)*p* = 0.003
—	—	—	—	Period 2 vs period 1 IRR (95% CI)^a^
All ages	—	304,378 (100.0)	388,144 (100.0)	1.27 (1.22, 1.33) *p* < 0.001
Daily admission rate	N/day Mean (SD)	N/day Mean (SD)	N/day Mean (SD)	Period 2 vs period 1 IRR (95% CI)^a^
18–35 years	29.6 (9.6)	26.2 (8.9)	33.0 (9.1)	1.26 (1.24, 1.28) *p* < 0.001
35–49 years	25.9 (9.5)	24.0 (9.2)	27.8 (9.4)	1.16 (1.14, 1.19) *p* < 0.001
50–64 years	32.7 (12.3)	29.5 (10.8)	35.9 (12.9)	1.22 (1.19, 1.24) *p* < 0.001
65–79 years	39.2 (15.3)	33.8 (12.4)	44.6 (16.1)	1.32 (1.29, 1.35) *p* < 0.001
80 + years	30.6 (11.0)	25.3 (8.6)	35.9 (10.5)	1.42 (1.39, 1.44) *p* < 0.001
All ages	31.6 (12.5)	27.8 (10.7)	34.4 (13.1)	1.28 (1.26, 1.29) *p* < 0.001
Admissions per patient	Mean (SD)	Mean (SD)	Mean (SD)	Period 2 vs Period 1 IRR (95% CI)^b^
18–35 years	1.85 (6.1)	1.8 (4.6)	1.9 (7.1)	1.047 (1.000, 1.098)*p* = 0.054
35–49 years	2.1 (9.7)	2.1 (10.2)	2.1 (9.3)	1.008 (0.932, 1.091)*p* = 0.836
50–64 years	2.6 (14.0)	2.6 (16.0)	2.6 (12.1)	0.981 (0.894,1.076)*p* = 0.679
65–79 years	3.1 (17.5)	3.2 (18.4)	3.0 (16.8)	0.957 (0.870, 1.053)*p* = 0.369
80 + years	3.3 (18.2)	3.2 (17.3)	3.5 (18.9)	1.095 (0.983, 1.220)*p* = 0.099
All ages	2.5 (13.3)	2.5 (13.7)	2.5 (13.1)	1.024 (0.984, 1.066)*p* = 0.243

N, Number; CI, Confidence Interval; IRR, Incidence rate ratio; SD, standard deviation.

^a^ Obtained using negative binomial regression and robust standard errors with age-group and period as fixed effects and the sum of all adult admissions each day as the dependent variable.

^b^ Obtained using negative binomial regression and robust standard errors with age-group and period as fixed effects and the sum of an individual patient’s admissions as the dependent variable.

Table 1 also shows the number of admissions per patient for each age group and study period. The highest rate of admissions per patient was for the 80+year-old age group with an overall mean (SD) of 3.3 (18.2) admissions per patient across the 12 years of the study. The number of admissions per patient was similar between the 2 study periods for all 5 age groups (*p* > 0.05 for each).

### Characteristics of the medicated and non-medicated population

Of the 692,522 admissions, 300,498 (43.4%) had at least one recorded medication (referred to as the medicated population) (Table 2). Supplementary Table S3 describes the CCI by age group and period for the medicated and non-medicated admissions separately. Amongst the medicated population, the mean (SD) for the CCI was 1.08 (1.69). The mean (SD) CCI ranged from 0.20 (0.70) for the 18–35-year-olds to 1.35 (1.72) for the 80 + years age-group. The mean CCI increased over time for all 5 age-groups with an overall increase across all ages of 20.3% (IRR = 1.203; 95% CI = 1.19, 1.217; *p* < 0.001).

**TABLE 2:** Medication usage by age-group and study period.

	All years	Period 1	Period 2	
	2007–2018	2007–2012	2013–2018	
	N = 692,522	N = 304,378	N = 388,144	
No. Of admissions with recorded medications, n (%)	N (%)	N (%)	N (%)	Period 2 vs period 1 IRR (95% CI)^a^ *p*-value
18–35 years	40,177 (31.0)	19,548 (34.05)	20,629 (28.5)	0.838 (0.822, 0.855)*p* < 0.001
35–49 years	47,387 (41.7)	23,541 (44.8)	23,846 (39.1)	0.873 (0.857, 0.889)*p* < 0.001
50–64 years	66,380 (46.3)	30,187 (46.6)	36,197 (46.0)	0.986 (0.971, 1.002)*p* = 0.080
65–79 years	77,518 (45.1)	32,875 (44.3)	44,643 (45.7)	1.032 (1.017, 1.047)*p* < 0.001
80 + years	69, 036 (51.5)	28,242 (50.9)	40,794 (51.9)	1.021 (1.005, 1.036)*p* = 0.009
All ages	300,498 (43.4)	134,410 (44.15)	166,105 (42.8)	0.969 (0.962, 0.976)*p* < 0.001
No. Medications for medicated admissions	Mean (SD)	Mean (SD)	Mean (SD)	IRR (95% CI)^b^ Period 2 vs Period 1 *p*-value
18–35 years	3.09 (2.45)	3.09 (2.30)	3.09 (2.58)	1.000 (0.983, 1.015)0.908
35–49 years	4.71 (3.91)	4.60 (3.79)	4.81 (4.02)	1.046 (1.030, 1.061)<0.001
50–64 years	6.55 (4.56)	6.21 (4.25)	6.83 (4.79)	1.100 (1.088, 1.112)<0.001
65–79 years	8.28 (4.76)	8.00 (4.46)	8.49 (4.96)	1.061 (1.053, 1.070)<0.001
80 + years	9.22 (4.48)	9.10 (4.34)	9.30 (4.57)	1.022 (1.015, 1.030)<0.001
All ages	6.86 (4.77)	6.52 (4.53)	7.13 (4.93)	1.093 (1.088, 1.099)<0.001
No. Medications for all admissions	Mean (SD)	Mean (SD)	Mean (SD)	IRR (95% CI)^b^ Period 2 vs Period 1 *p*-value
18–35 years	0.96 (1.97)	1.05 (1.99)	0.88 (1.96)	0.837 (0.819, 0.853)<0.001
35–49 years	1.96 (3.43)	2.06 (3.42)	1.88 (3.44)	0.913 (0.895, 0.932)<0.001
50–64 years	3.03 (4.50)	2.89 (4.25)	3.14 (4.70)	1.085 (1.068, 1.102)<0.001
65–79 years	3.74 (5.22)	3.54 (4.96)	3.88 (5.40)	1.095 (1.081, 1.110)<0.001
80 + years	4.75 (5.62)	4.63 (5.50)	4.83 (5.70)	1.043 (1.030, 1.057)<0.001
All ages	2.97 (4.63)	2.88 (4.42)	3.05 (4.78)	1.060 (1.052, 1.068)<0.001

N, Number; CI, Confidence Interval; IRR, Incidence rate ratio; SD, standard deviation.

^a^ Using negative binomial regression and robust standard errors with period as the independent variable and medicated/non-medicated status as the dependent variable.

^b^ Using negative binomial regression with period as the independent variable and the number of medications recorded for each admission as the dependent variable.

Amongst the non-medicated admissions, the mean (SD) CCI was slightly lower than the CCI for the medicated admissions both for the overall population (0.88 (1.18)) and for each of the 5 age groups. In addition, and in contrast to the medicated admission population, the CCI was lower in the second period of the study for each age-group (*p* < 0.05 for each) except for the 18–35-year-olds, where the CCI increased (IRR = 1.091; 95% CI = 1.041, 1.143; *p* < 0.001).

Supplementary Figure S2 shows the change in the prevalence over time for each of the 17 conditions that contribute to the CCI. The main driver of the change in the CCI was for diabetes and diabetes with complications, which fell between 2007 and 2011 and then increased again. Most of the other comorbidities remained relatively stable over time.

Supplementary Table S4 shows the proportion of males and females making up the medicated and the non-medicated admissions by age group and period. Overall, the proportion of males and females that were medicated was similar (49.6% *versus* 50.4%). However, there were relatively more females than males in the medicated admission population for age-groups 18–35 years, 35–49 years, and 80 + years of age. Amongst the non-medicated admissions, 45.3% were male and 54.7% were female overall. However, the proportion of males and females that were non-medicated varied widely across age groups and was as high as 80.6% for females (19.4% for males) in the 18–35-year-old age group. Amongst all female admissions in the 18–35-year-old age group, only *n* = 22,283 (23.6%) of all 94,402 admissions were medicated compared with males in the same age group for which n = 17,894 (50.7%) of the *n* = 35,285 admissions were medicated.

### Drug class usage

**FIGURE 1:**
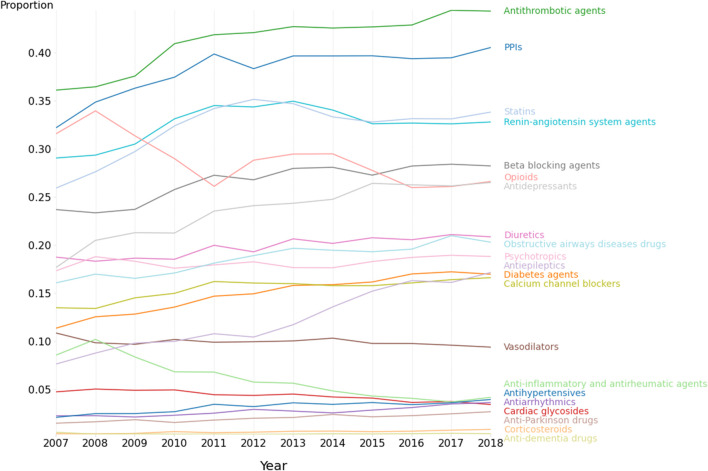
Medication class use amongst patients with recorded medications. 2007–2018. N = 692,522 admissions.

Figure 1 shows the overall prevalence of drug class use amongst the medicated patient admissions for the 21 specified drug classes, and for each year. In 2018, the 10 highest rates of drug class usage were for antithrombotic agents (44.3% of all medicated admissions), proton pump inhibitors (40.6%), statins (33.8%), renin-angiotensin system agents (32.8%), beta-blocking agents (28.2%), opioids (26.6%), antidepressants (26.5%), diuretics (20.9%), obstructive airways disease drugs (20.3%) and psychotropics (18.8%).

**FIGURE 2:**
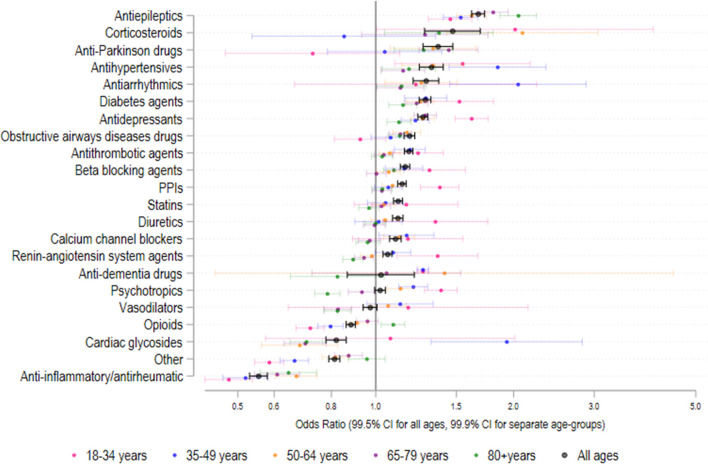
Odds ratio (95% CI) in medication class usage 2013–2018 versus 2007–2012 amongst patients with recorded medication use. N = 692,522 admissions.

Figure 2 shows both the overall odds ratio (OR) for usage of each specified drug class for period 2 (2013–2018) *versus* period 1 (2007–2012), and the corresponding age-group specific odds ratios. The odds ratio [and corresponding 99.5% CI] between periods 1 and 2 were greater than 1.00 (indicating *p* < 0.005) for antiepileptics; OR = 1.67 [1.62–1.73], corticosteroids; OR = [1.47; [1.29–1.69], anti-Parkinson drugs; OR = 1.37 [1.27–1.47], antihypertensives; OR = 1.32 [1.24–1.40], diabetes agents; OR = 1.28 [1.24–1.32], antidepressants; OR = 1.27 [1.24–1.30], obstructive airways disease drugs; OR = 1.18 [1.15–1.22], antithrombotic agents; OR = 1.18 [1.16–1.21], beta-blocking agents; OR = 1.16 [1.13–1.19], proton pump inhibitors; OR = 1.14 [1.12–1.17], statins; OR = 1.12; [1.09–1.14], diuretics; OR = 1.12; [1.09–1.15], calcium channel blockers; OR = 1.10 [1.07–1.14), and renin-angiotensin system agents; OR = 1.06 [1.04–1.08]. As shown in Figure 1, the medication use for some classes, e.g., proton pump inhibitors, statins, renin-angiotensin system agents, tended to plateau or even decrease during period 2. Of the 21 different drug classes considered, 3 decreased (OR and 99.5% CI < 1.00, *p* < 0.005) between periods 1 and 2 including opioids; OR = 0.88 [0.86–0.90], cardiac glycosides; OR = 0.82 [0.78–0.86], Other agents; OR = 0.81 [0.79–0.83] and anti-inflammatory/antirheumatic drugs; OR = 0.55 [0.53–0.58].

### Mean medication usage

**FIGURE 3:**
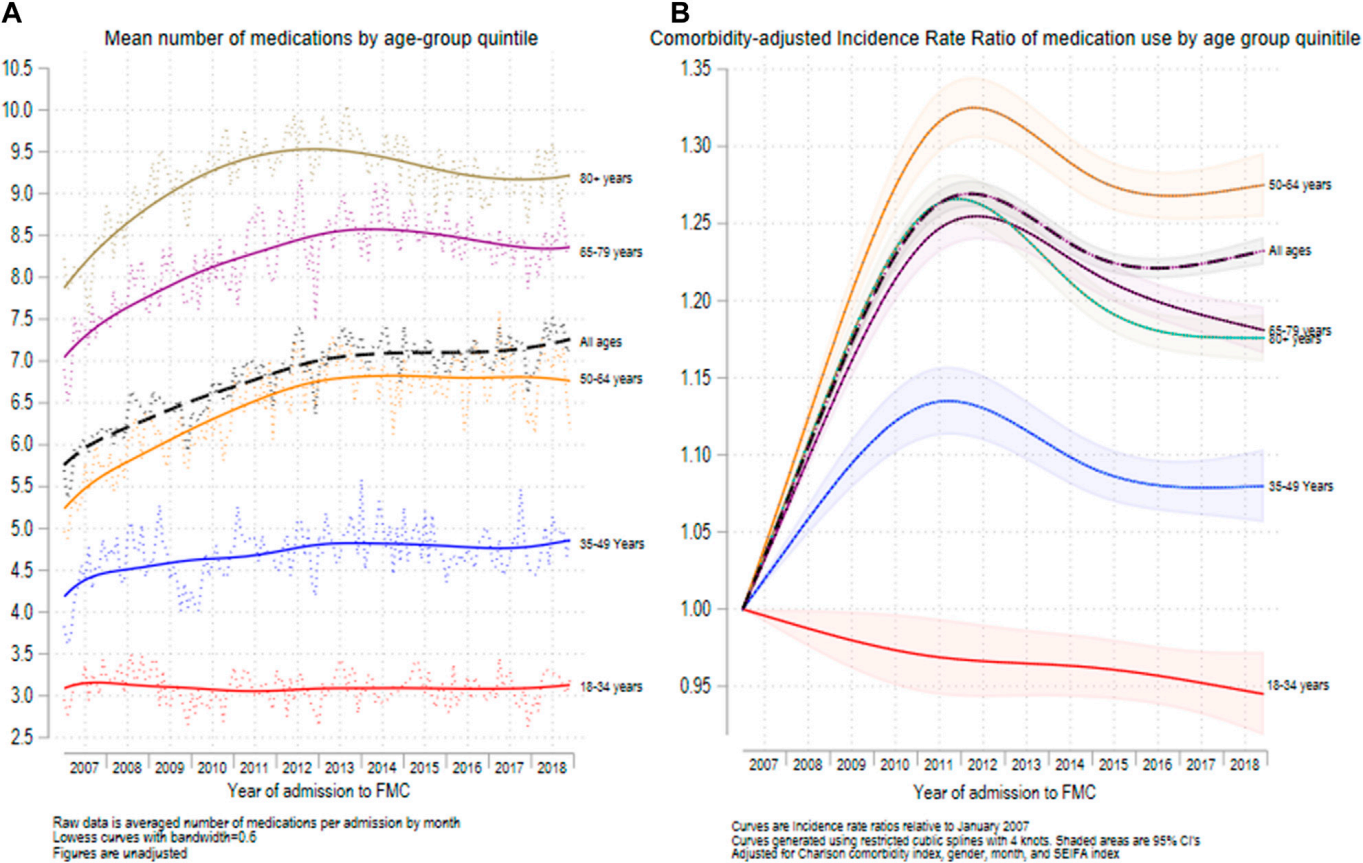
Mean number of medications recorded **(** A **)** and relative increase in medications recorded since 2007 **(** B **)** for the 5 major age groups. N = 692,522 admissions.

Figures 3A,B show the mean number of medications for medicated hospital admissions (Figure 3A) and the incidence rate ratio of medication usage for each year relative to 2007 (Figure 3B), for each age group across the 12 years of the study. In unadjusted analysis, the mean number of medications per medicated admission did not change significantly between periods 1 and 2 for the 18-to-35-year-old age group (IRR = 1.000; 95% CI = 0.983–1.015; *p* < 0.001) (Figure 3A and Table 2). However, the mean (SD) number of medications increased significantly between periods 1 and 2 for each of the other 4 age groups (Table 2). Overall, the mean (SD) number of medications for medicated admissions increased from 5.93 (4.04) in 2007 to 7.21 (4.98) in 2018. Restricted cubic spline analysis (Figure 3B) showed that the mean number of medications for all admissions increased between 2007 and 2011 and then declined until 2018 for each age group, except for the 18-to-35-year-olds in which there was a gradual decline in the number of medications from 2007 until 2018.

Supplementary Figure S3 shows the results of non-linear regression to detect changes in linear trends for medication use amongst the medicated admissions. After adjustment for age, sex, month, and CCI, there was a significant linear increase (*β* = 0.296; 95% CI = 0.269, 0.323, *p* < 0.001) in mean medications throughout the first period of the study and a significant decrease (*β* = -0.070; 95% CI = -0.058, -0.082, *p* < 0.001) in the second period of the study. The change in slopes was also significant (*p* < 0.001).

### Specific medication usage

**FIGURE 4:**
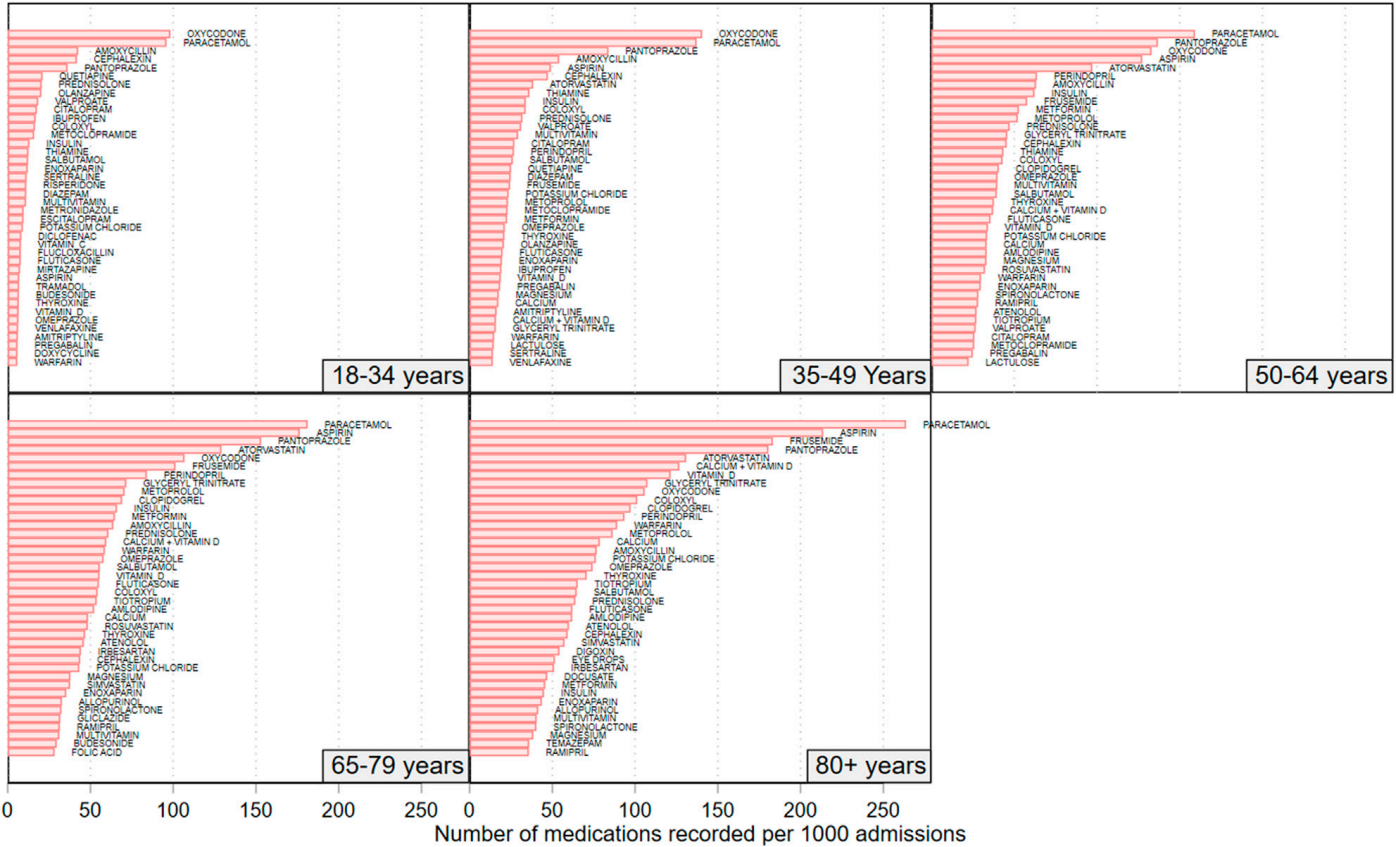
Rate of recorded medications for the top 40 recorded medications amongst patients in each of the studies 5 age groups. N = 692,522 admissions.

Figure 4 shows the medication usage rate for the 40 highest-use individual medications for each of the 5 age groups. Amongst the 18–34-year-olds, the 5 highest recorded medication rates (number per 1,000 admissions) were for oxycodone (98.2), paracetamol (95.9), amoxicillin (42.6), pantoprazole (36.1), and cefalexin (21.3). Among the 35–49-year-olds, the highest rates were for oxycodone (140.5), paracetamol (137.2), pantoprazole (83.9), amoxicillin (54.1), and aspirin (49.1). Among the 50-to-64-year-olds, the highest recorded rates were for paracetamol (159.2), pantoprazole (136.8), oxycodone (133.0), aspirin (127.3), and atorvastatin (97.2). In the 65-to-79-year-olds, the highest recorded rates were for paracetamol (182.2), aspirin (176.4), pantoprazole (153.1), atorvastatin (129.2), and oxycodone (106.8). Among the 80+ year-olds, the highest recorded rates were for paracetamol (263.7), aspirin (213.6), frusemide (183.3), pantoprazole (180.6), and atorvastatin (130.8).

Supplementary Figure 4 shows the medications with the highest recorded medication rates for adult patients aged less than 65 years of age and adult patients aged 65 + years of age. Amongst those aged less than 65 years of age, the 5 most frequently recorded medications were for paracetamol (131.5/1,000 admissions), oxycodone (123.5/1,000 admissions), pantoprazole (87.5/1,000 admissions), aspirin (64.0/1,000 admissions), and amoxicillin (53.6/1,000 admissions). Amongst the patients aged 65 and years and older, the 5 most frequently recorded admissions were for paracetamol (217.4/1,000 admissions), aspirin (192.7/1,000 admissions), pantoprazole (165.1/1,000 admissions), frusemide (137.2/1,000 admissions) and atorvastatin (129.9/1,000 admissions).

Supplementary Figure S5 shows the medications with the highest recorded medication rates for adult patients admitted between 2007 and 2012 and for those admitted between 2013 and 2018. Amongst those admitted 2007–2012, the 5 most frequently recorded medications (number per 1,000 admissions) were for paracetamol (195.7/1,000 admissions), oxycodone (133.6/1,000admissions), aspirin (127.3/1,000 admissions), pantoprazole (120.1/1,000 admissions), and atorvastatin (84.3/1,000 admissions). Amongst patients admitted 2013–2018, the 5 most frequently recorded medications were for paracetamol (148.9/1,000 admissions), pantoprazole (123.1/1,000 admissions), aspirin (115.8/1,000 admissions), oxycodone (102.1/1,000 admissions) and atorvastatin (84.5/1,000 admissions).

## Discussion

In this study, we report that the use of electronic hospital admission data can robustly capture temporal trends in medication usage for a wide range of age groups, drug classes, and comorbidity burden in a local Australian health network. We observed significantly higher admission rates for all age groups for 2013–2018 than 2007–2012, with the largest increase being for the 65–79 years’ group. A similar temporal increase in the CCI was observed for all age groups in the medicated, but not in the non-medicated, study population. Overall, the total number of medications per medicated admission significantly increased during the 12-year observation period for all age groups, barring the 18–35 years’ group. This phenomenon might be accounted for, at least partly, by the concomitant increase in the CCI, reflecting higher patient complexity and comorbidity burden. However, it is important to highlight that the observed increase in the number of medications during period 1 (2007–2012) was followed by a gradual decline for most age groups during period 2 (2013–2018). Different temporal trends were observed with specific drug classes. A significant overall increase in the rate of medication use between period 1 and period 2 was observed for antithrombotic agents, proton pump inhibitors, statins, renin-angiotensin system agents, beta-blocking agents, antidepressants, diuretics, obstructive airways disease drugs, anti-Parkinson drugs, corticosteroids, and antiepileptics. By contrast, a significant reduction in the rate of medication use between period 1 and period 2 was observed for opioids, cardiac glycosides, and anti-inflammatory/antirheumatic drugs. In addition to these overall changes between the first period (2017–2012) and second period (2013–2018) of the study, there were several noteworthy temporal trends. The use of proton pump inhibitors and statins stabilised between 2013 and 2017, the use of renin-angiotensin system agents declined between 2013 and 2015 and then stabilised to 2018, the use of cardiac glycosides gradually declined from 2010 to 2018, opiod use declined between 2008 and 2016, and beta-blocker use increased steadily between 2008.

Hospital data have been increasingly used, particularly with the availability of electronic medical records, in pharmacoepidemiologic studies. Significant differences with other databases, i.e., primary care and community databases, include the use of highly specific agents and formulations (e.g., highly potent, and broad-spectrum antibiotics given intravenously) and the higher frailty and co-morbid burden of hospitalized patients. A review published in 2013 identified a relatively small number, twelve, of hospital inpatient databases used for pharmacoepidemiologic studies in Europe, the USA, and Asia. The majority of the databases were developed in university hospitals and investigated a wide range of issues, e.g., drug utilization practices, costs, safety, and pharmacovigilance, using prescription data during hospitalization (Larsen et al., 2013). More recently, hospital discharge data have been used in Australia and other countries to identify patients at high risk of adverse drug reactions post-discharge and readmission (Nguyen et al., 2016; Fanning et al., 2018). Our study extends the potential applications of hospital data for pharmacoepidemiologic research, focusing on information regarding medication use and a wide range of patient characteristics at the time of admission to investigate temporal trends in medication usage in the community.

The observed temporal increase in the use of specific drug classes is in line with the results of other studies conducted in Australia and/or elsewhere on antithrombotic agents (Apenteng et al., 2018; Bezabhe et al., 2021b), proton pump inhibitors (Hollingworth et al., 2010; Abrahami et al., 2020), statins (O'Keeffe et al., 2016; Ofori-Asenso et al., 2018), beta-blockers (Uijl et al., 2021), and antidepressants (Alduhishy, 2018; de Oliveira Costa et al., 2022). A relative plateauing in use in more recent years has also been reported for proton pump inhibitors (Pratt et al., 2017; Abrahami et al., 2020), and statins (O'Keeffe et al., 2016; Ofori-Asenso et al., 2018). For proton pump inhibitors, national initiatives, e.g., the Australian Government Department of Veterans’ Affairs Veteran’s Medicines Advice and Therapeutics Education Services (MATES) and the National Prescribing Service MedicineWise programs, and local interventions have likely led to a decrease in their inappropriate use particularly in the older population (Hamzat et al., 2012; Pratt et al., 2017; Wilsdon et al., 2017). The relatively lower use of statins in more recent years could be attributed to changes in professional guidelines, increasing awareness of potential side effects, and availability of alternative lipid-lowering agents (O'Keeffe et al., 2016; Ofori-Asenso et al., 2018). The observed temporal reduction in the use of cardiac glycosides has also been observed in other studies, possibly reflecting the increasing availability of alternative agents for the management of atrial fibrillation and heart failure (Patel et al., 2016; Bezabhe et al., 2021a). By contrast, the reduction in the use of opioids is in contrast with the results of a systematic review reporting a significant increase in opioid prescribing in Australia from the 1990s to 2017 (Donovan et al., 2020), suggesting that local policies and prescribing practices might differ from overall national trends.

Another important finding in our study was the gradual decline in the total number of medications per medicated admission during the second half of the study period. In line with our results, an Australian study investigating national trends in polypharmacy, defined as the use of ≥5 medications by an individual patient, between 2006 and 2017, has reported a decline in the years 2016 and 2017 (Page et al., 2019). The authors suggest possible reasons for this finding, including policy changes affecting the supply of medicines listed in the national Prescribing Benefits Scheme, which provides subsidized access to prescribed drugs to Australian citizens and permanent residents. However, another Australian study investigating trends in medication use in the older population also suggests the presence of significant variability in trajectories across different sub-groups, i.e., sustained polypharmacy vs decreasing medication use (Falster et al., 2021). Clearly, more research is warranted to identify the factors responsible for the different patterns of medication use and polypharmacy in Australia and worldwide.

Pending the development and implementation of robust state, territory, and national Australian data linkage systems combining prescribing information with other patient characteristics and health outcomes, the results of this study support the proposition that electronic patient admission data can be useful to monitor local trends in medication use. The reliability of this information is supported by the fact that the observed trends in medication use are in line with those reported in other studies conducted in Australia and overseas. At the same time, discordant data, e.g., with opioid medications, might provide useful insights into different policies and prescribing practices across health networks. Specific advantages of using electronic hospital data include the process of medication reconciliation on admission adopted by most Australian public hospitals, which ensures that the information regarding the type and the dose of medications is checked against different sources (Stark et al., 2020). Furthermore, data from tertiary referral centers generally allow the assessment of a wide range of age groups and comorbidity burden, unlike registries that capture specific cohorts, e.g., residents in aged care facilities and Australian Veterans (Mangoni et al., 2010a; Mangoni et al., 2010b; Ambagtsheer et al., 2020). It is also possible to link hospital admission data with clinical outcomes such as short- and mid-term mortality and readmission rates (Bryant et al., 2019). Potential limitations also need to be acknowledged. In particular, medication changes during hospitalization, including targeted deprescribing strategies (Tay et al., 2014; Russell et al., 2019; Kaminaga et al., 2021; Roberts et al., 2021; Russell et al., 2021; Scott et al., 2021), inevitably lead to deviations from the medication list on admission. This, however, could be rectified by additional linkage to discharge and/or readmission data. Regarding the accuracy and consistency of the hospital admission data over time, we examined changes in the Charlson comorbidity Index (CCI) over time since one might expect that to see this variable stay relatively stable over time. Of note however, within the 18–35-year-old age group of non-medicated patients, the Charlson comorbidity index, although low (0.16–0,17) at baseline as would be expected, increased by 9.1% between periods (IRR = 1.091 (1.041–1.143, *p* < 0.001)). This modest increase from a low baseline level either reflect a regression to the mean effect, with a somewhat lower than usual value in the first 6-year of the study, or alternatively it may signal that there was a real slight increase in the presence of previously undiagnosed chronic disease which has not yet been treated.

Strengths of our study include the assessment of a large and unselected study population admitted to a large metropolitan tertiary teaching hospital over a period of 12 years, the study of a wide range of medication classes, and the evaluation of the impact of age and of robust measures of comorbidity on temporal medication trends. In addition, the large dataset provided sufficient power to allow stratification of the analysis by commonly defined age groups, and a thorough examination of the trends over time using several different statistical approaches. Limitations include the lack of information on measures of frailty, cognitive and physical function, potentially affecting medication use *per se*, the single-center nature of the study, and the potential for measurement error and residual confounding. However, it is unlikely that any measurement error for medications would have differed significantly over time since there were no changes in recording protocols. Similarly, the case-mix of patients presenting to the hospital is also unlikely to have differed over the study period given that there were no major changes in health services provision in the geographical catchment area of the study. The finding that our results largely reflect medication trends observed in other Australian and international studies also supports the validity of the collected data and suggests that electronic hospital admission data adequately reflect “real-world” practices in medication use.

In conclusion, our study suggests that the use of electronic hospital admission data can assist with the monitoring of local trends in the use of medications and the effects of initiatives to enhance the quality use of medicines and reduce the burden of inappropriate prescribing.

## Data Availability

The original contributions presented in the study are included in the article/supplementary material. Further inquiries can be directed to the corresponding author: arduino.mangoni@flinders.edu.au.
