# Supplementary material for: Using electronic admission data to monitor temporal trends in local medication use: Experience from an Australian tertiary teaching hospital
Source: Front Pharmacol. 2022 Oct 14;13:888677. doi: 10.3389/fphar.2022.888677 (PMC9614045; doi:10.3389/fphar.2022.888677)
Supplement: Supplementary file 1 [file Table1.docx]

**Suppl Table1**: Anatomical Therapeutic Chemical (ATC) drug class codes of the 21 classes of drugs analysed.

| **Drug Class** | **ATC Code** |
| --- | --- |
| PPIs | A02BC |
| Diabetes | A10A, A10B |
| Antithrombotic agents | B01A |
| Cardiac glycosides | C01A |
| Antiarrhythmics | C01B |
| Vasodilators | C01D |
| Diuretics | C03A, C03B, C03C, C03D, C03E, C03X |
| Beta blocking agents | C07A |
| Calcium channel blockers | C08C, C08D, C08E, C08G |
| Renin-angiotensin system agents | C09A, C09B, C09C, C09D, C09X |
| Statins | C10AA |
| Corticosteroids | H02A, H02B |
| Anti-inflammatory and antirheumatic agents | M01A |
| Opioids | N02A |
| Antiepileptics | N03A |
| Anti-Parkinson drugs | N04A, N04B, N04C |
| Psychotropics | N05A, N05B, N05C |
| Antidepressants | N06A |
| Anti-dementia drugs | N06D |
| Obstructive airways diseases drugs | R03A, R03B, R03C, R03D |
| Antihypertensives | C02A, C02B, C02C, C02D, C02K, C02L, C02N |

**Suppl Table 2:** Patient demographics and clinical characteristics for Period 1 (2007-2012) and Period 2 (2013-2018)

|  | 2007-2012  N=304,378 | 2013-2018  N=388,144 | p-value^1^ |
| --- | --- | --- | --- |
| Age  Mean (SD)  Median (IQR) | 57.57 (20.75)  60.0 (40.0-76.0) | 58.76 (21.08)  62.0 (40.0-77.0) | <0.001  <0.001 |
| Gender  Male, n (%)  Female, n (%) | 144,466 (47.5)  159, 912 (52.5) | 182, 044 (46.9)  206, 100 (53.1) | <0.001 |
| SEIFA  Mean (SD)  SEIFA<1000  SEIFA≥1000  MIssing | 981.9 (63.1)  193, 878 (63.70)  109, 827 (36.08)  673 (0.22) | 981.5 (62.1)  248, 002 (63.89)  139, 152 (35.85)  990 (0.26) | 0.011  0.003 |
| Charslon co-morbidity Index  Mean (SD)  Median (IQR)  1 or 2 (Mild)  3 or 4 (Moderate)  ≥5 (Severe) | 0.93 (1.41)  0 (0, 2)  178, 227 (58.55)  27, 132 (8.9)  99, 019 (32.5) | 0.995 (1.44)  0 (0, 2)  214,073 (55.15)  45,795 (11.8)  128, 276 (33.05) | <0.001  <0.001  <0.001 |
| Length of hospital stay (Days)  Mean (SD)  Median (IQR) | 3.71 (7.30)  1 (1, 3) | 3.27 (5.91)  1 (1, 3) | <0.001 |
| Died in hospital, n (%)  No  Yes | 300,597 (98.76)  3,781 (1.24) | 384, 107 (98.96)  4,037 (1.04) | <0.001 |
| Discharged to RACF, n (%)  No  Yes | 301, 370 (99.01)  3,008 (0.99) | 384,988 (99.19)  3,156 (0.81) | <0.001 |
| Listed ICD-10 codes^2^, n (%)  Infectious Disease  Hepatitis B  Malignancy  Endocrine Disease  Use of Marijuana  Dementia/Alzheimers  Neurological  Cardiovascular Disease  Respiratory Disease  Digestive disturbances  Rheumatic diseases  Chronic renal failure | 23,189 (7.62)  1,713 (0.56)  27,074 (8.89)  37,205 (12.22)  2,202 (0.72)  4,029 (1.32)  19,430 (6.38)  57,086 (18.75)  26,746 (8.79)  35,820 (11.77)  18,430 (6.05)  9,577 (3.15) | 38,918 (10.03)  5,712 (1.47)  33,513 (8.63)  83,271 (21.45)  2,422 (0.62)  4,691 (1.21)  22,318 (5.75)  68,177 (17.56)  37,162 (9.57)  48,014 (12.37)  23,823 (6.14)  13,218 (3.41) | <0.001  <0.001  <0.001  <0.001  <0.001  <0.001  <0.001  <0.001  <0.001  <0.001  <0.001  <0.001 |

SEIFA=Socioeconomic Indexes for Areas. RACF=Residential Aged Care Facility. ICD-10=International Classification of Diseases 10^th^ revision.

^1^Using t-test, mann-whitney test or chi-squared test

^2^Based on either the Primary diagnosis code or one of up to 24 secondary diagnosis codes

**Suppl Table 3:** Charlson comorbidity index by age-group and study period amongst medicated and non-medicated admissions (n=692,522).

|  | **All years** | **Period 1** | **Period 2** | **Period 2 vs Period 1**  **IRR^1^ (95% CI)** |
| --- | --- | --- | --- | --- |
| **Medicated admissions** | **2007-2018**  **N=300,498** | **2007-2012**  **N=134,393** | **2013-2018**  **N=166,105** |  |
| Age-group  18-35 years | Mean (SD)  0.20 (0.70) | Mean (SD)  0.18 (0.69) | Mean (SD)  0.22 (0.71) | 1.190 (1.110, 1.275)  <0.001 |
| 35-49 years | 0.61 (1.32) | 0.55 (1.31) | 0.67 (1.34) | 1.201 (1.155, 1.250)  <0.001 |
| 50-64 years | 1.19 (1.79) | 1.07 (1.76) | 1.30 (1.82) | 1.219 (1.190, 1.248)  <0.001 |
| 65-79 years | 1.48 (1.91) | 1.37 (1.89) | 1.56 (1.91) | 1.136 (1.115, 1.158)  <0.001 |
| 80+ years | 1.35 (1.72) | 1.29 (1.72) | 1.39 (1.71) | 1.081 (1.060, 1.103)  <0.001 |
| All ages | 1.08 (1.69) | 0.97 (1.66) | 1.17 (1.72) | 1.203 (1.190, 1.217)  <0.001 |
|  |  |  |  |  |
| **Non-medicated admissions** | **2007-2018**  **N=392,024** | **2007-2012**  **N=169,985** | **2013-2018**  **N=222,039** | **Period 2 vs Period 1 IRR^1^ (95% CI)** |
| Age-group  18-35 years | Mean (SD)  0.16 (0.57) | Mean (SD)  0.16 (0.57) | Mean (SD)  0.17 (0.57) | 1.091 (1.041, 1.143)  <0.001 |
| 35-49 years | 0.56 (1.03) | 0.56 (1.05) | 0.55 (1.01) | 0.971 (0.944, 0.999)  0.045 |
| 50-64 years | 1.10 (1.26) | 1.14 (1.26) | 1.06 (1.26) | 0.930 (0.915, 0.945)  <0.001 |
| 65-79 years | 1.30 (1.24) | 1.33 (1.21) | 1.28 (1.26) | 0.964 (0.952, 0.975)  <0.001 |
| 80+ years | 1.34 (1.20) | 1.36 (1.16) | 1.33 (1.22) | 0.981 (0.968, 0.995)  <0.001 |
| All ages | 0.88 (1.18) | 0.90 (1.18) | 0.87 (1.18) | 0.959 (0.951, 0.967)  <0.001 |

N=Number. CI=Confidence Interval. IRR=Incidence rate ratio. SD=Standard deviation.

^1^Using negative binomial regression with robust standard errors and the period (period 2 versus period 1) as the independent variable.

**Suppl Table 4:** Gender breakdown by age-group and study period amongst medicated and non-medicated adult admissions.

|  | **All years** | **Period 1** | **Period 2** | **OR (95% CI) for male gender Period 2 vs Period 1^1^ (p-value)** |
| --- | --- | --- | --- | --- |
|  | **2007-2018**  **N=300,498** | **2007-2012**  **N=134,393** | **2013-2018**  **N=166,105** |  |
| **Medicated admissions** | Males/Females  N (%) | Males/Females  N (%) | Males/Females  N (%) |  |
| 18-35 years | 17,894/22,283  (44.5/55.5) | 9,208/10,340  (47.1/52.9) | 8,686/11,943  (42.1/57.9) | 0.82 (0.79, 0.85)  P<0.001 |
| 35-49 years | 22,649/24,738  (47.8/52.2) | 11,268/12,273  (47.9/52.1) | 11,381/12,465  (47.7/52.3) | 0.99 (0.96, 1.03)  P=0.763 |
| 50-64 years | 36,578/29,802  (55.1/44.9) | 16,553/13,634  (54.8/45.2) | 20,025/16,168  (55.3/44.7) | 1.02 (0.99, 1.05)  P=0.203 |
| 65-79 years | 41,504/36,014  (53.5/46.5) | 17,308/15,567  (52.65/47.35) | 24,196/20,447  (54.2/45.8) | 1.06 (1.03, 1.10)  P<0.001 |
| 80+ years | 30,346/38,690  (44.0/56.0) | 11,886/16,356  (42.1/57.9) | 18,460/22,334  (45.25/54.75) | 1.14 (1.10, 1.17)  P<0.001 |
| All ages | 148,971/151,527  (49.6/50.4) | 66,223/68,170  (49.3/50.7) | 82,748/83,357  (49.8/50.2) | 1.02 (1.01, 1.04)  P=0.003 |
|  |  |  |  |  |
|  | **2007-2018**  **N=392,024** | **2007-2012**  **N=169,985** | **2013-2018**  **N=222,039** | **OR (95% CI) for male gender Period 2 vs Period 1^1^ (p-value)** |
| **Non-medicated admissions** | **Males/Females**  **N (%)** | **Males/Females**  **N (%)** | **Males/Females**  **N (%)** |  |
| 18-35 years | 17,391/72,119  (19.4/80.6) | 6,991/30,866  (18.5/81.5) | 10,400/41,253  (20.1/80.0) | 1.11 (1.08, 1.15)  P<0.001 |
| 35-49 years | 25,099/41,072  (37.9/62.1) | 11,485/17,538  (39.6/60.4) | 13,614/23,534  (36.65/63.35) | 0.88 (0.86, 0.91)  P<0.001 |
| 50-64 years | 44,482/32,554  (54.7/42.3) | 20,513/14,031  (59.4/40.6) | 23,969/18,523  (56.4/43.6) | 0.89 (0.86, 0.91)  P<0.001 |
| 65-79 years | 52,827/41,432  (56.0/44.0) | 23,589/17,702  (57.1/42.9) | 29,238/23,730)  55.2/44.8 | 0.92 (0.90, 0.95)  <0.001 |
| 80+ years | 37,740/27,308  (58.0/42.0) | 15,665/11,605  (57.4/42.6) | 22,075/15,703  (58.4/41.6) | 1.04 (1.01, 1.07)  P=0.012 |
| All ages | 177,539/214,485  (45.3/54.7) | 78,243/91,742  (46.0/54.0) | 99,296/122,743  (44.7/55.3) | 0.95 (0.94, 0.96)  P<0.001 |

N=Number. CI=Confidence Interval. OR=Odds ratio.

^1^Using logistic regression with robust standard errors and period as the independent variable, stratified by age-group.
