# Supplementary figures and images for: Using electronic admission data to monitor temporal trends in local medication use: Experience from an Australian tertiary teaching hospital
Source: Front Pharmacol. 2022 Oct 14;13:888677. doi: 10.3389/fphar.2022.888677 (PMC9614045; doi:10.3389/fphar.2022.888677)

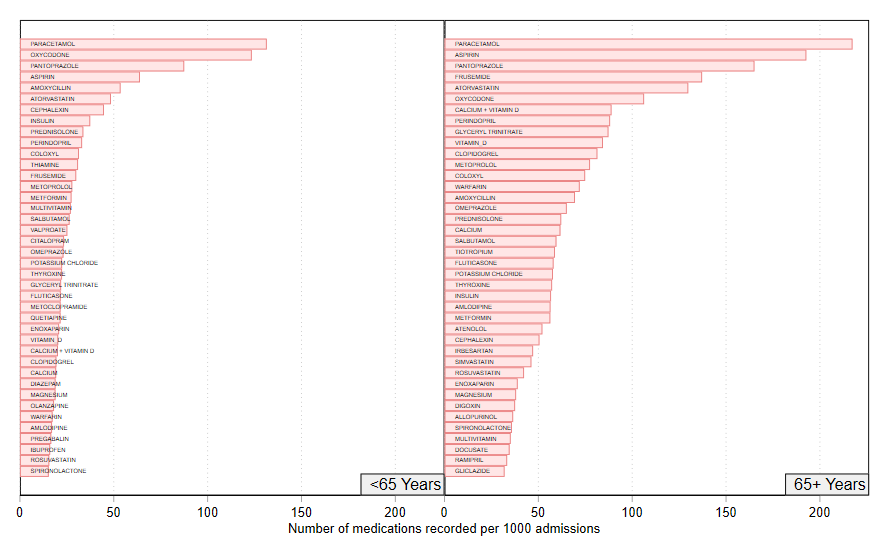

Supplement: Supplementary file 2 [file Image3.tif]

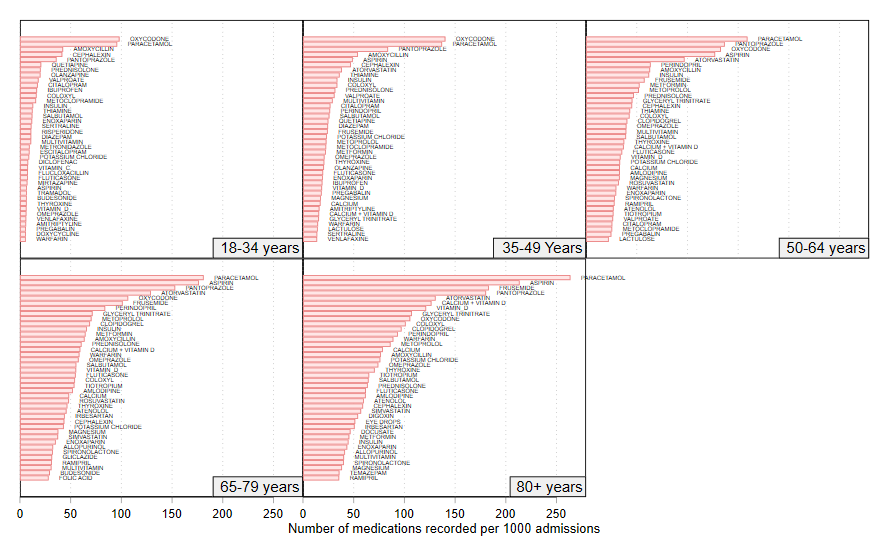

Supplement: Supplementary file 3 [file Image4.tif]

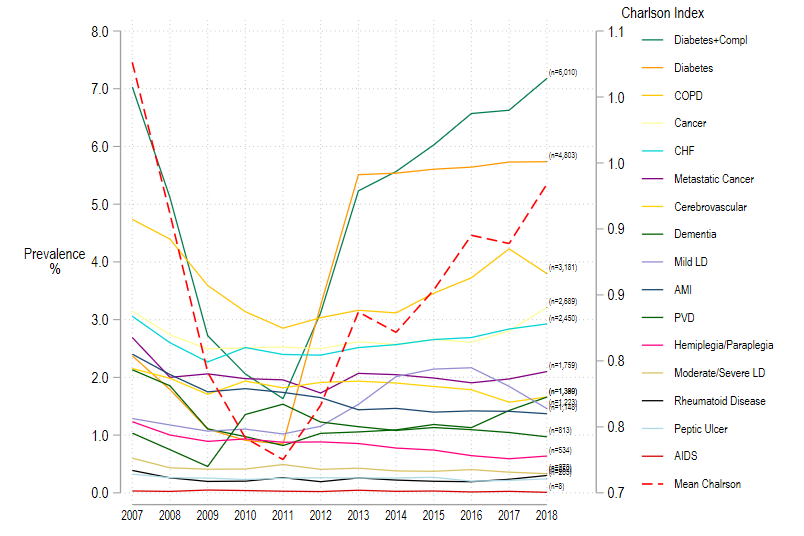

Supplement: Supplementary file 4 [file Image2.tif]

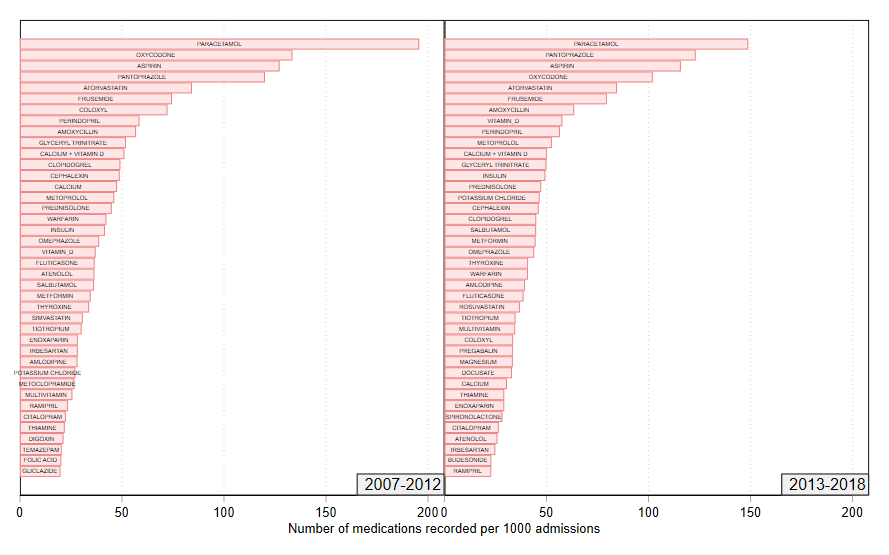

Supplement: Supplementary file 5 [file Image5.tif]
