# Supplementary material for: Using electronic admission data to monitor temporal trends in local medication use: Experience from an Australian tertiary teaching hospital
Source: Front Pharmacol. 2022 Oct 14;13:888677. doi: 10.3389/fphar.2022.888677 (PMC9614045; doi:10.3389/fphar.2022.888677)
Supplement: Supplementary file 6 [file Image1.pdf]

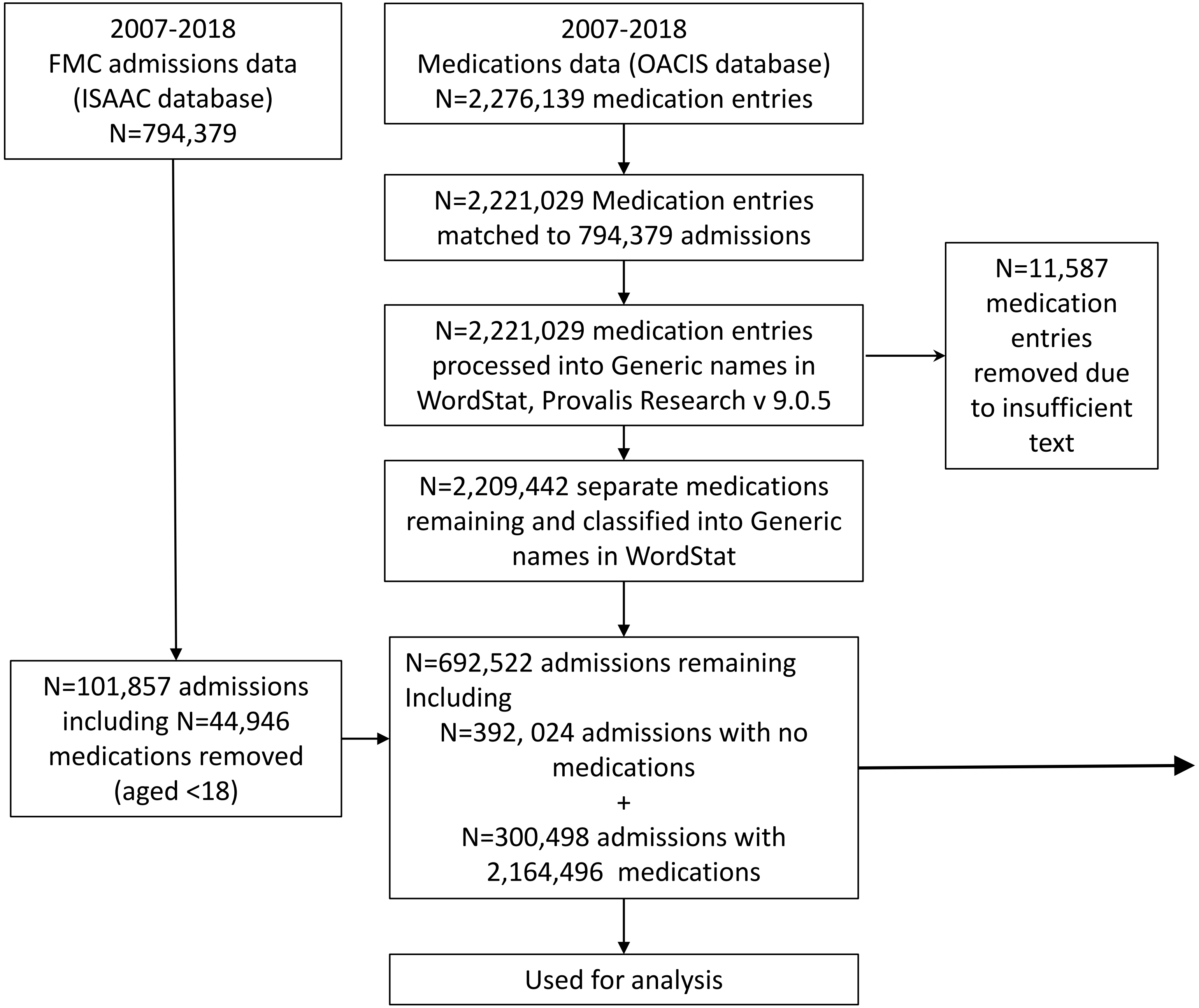

**Drug Class**	**N**	**%**
Other	860,331	39.75
Antithrombotic agents	151,585	7.00
Obstructive airways disease drugs	119,124	5.50
PPIs	118,465	5.47
Opioids	105,909	4.89
Renin-angiotensin system agents	102,035	4.71
Statins	98,907	4.57
Beta-blocking agents	84,979	3.93
Antidepressants	84,688	3.91
Diabetes	80,025	3.70
Diuretics	80,025	3.66
Psychotropics	78,471	3.63
Antiepileptics	50,314	2.32
Calcium channel blockers	49,849	2.30
Vasodilators	37,273	1.72
Anti-inflammatory and antirheumatic agents	19,334	0.89
Cardiac glycosides	13,192	0.61
Antihypertensives	10,776	0.50
Antiarrhythmics	9,168	0.42
Anti-Parkinson drugs	7,784	0.36
Corticosteroids	1,875	0.09
Anti-dementia drugs	1,166	0.05
**Total**	**2,164,496**	**100.00**
